# Supplementary material for: Individual and Group-Based Effects of In Vitro Fiber Interventions on the Fecal Microbiota
Source: Microorganisms. 2023 Aug 3;11(8):2001. doi: 10.3390/microorganisms11082001 (PMC10459671; doi:10.3390/microorganisms11082001)
Supplement: Supplementary file 1 [file microorganisms-11-02001-s001.zip › microorganisms-2526744-supplementary/SupplementaryFigureS1a.pdf]

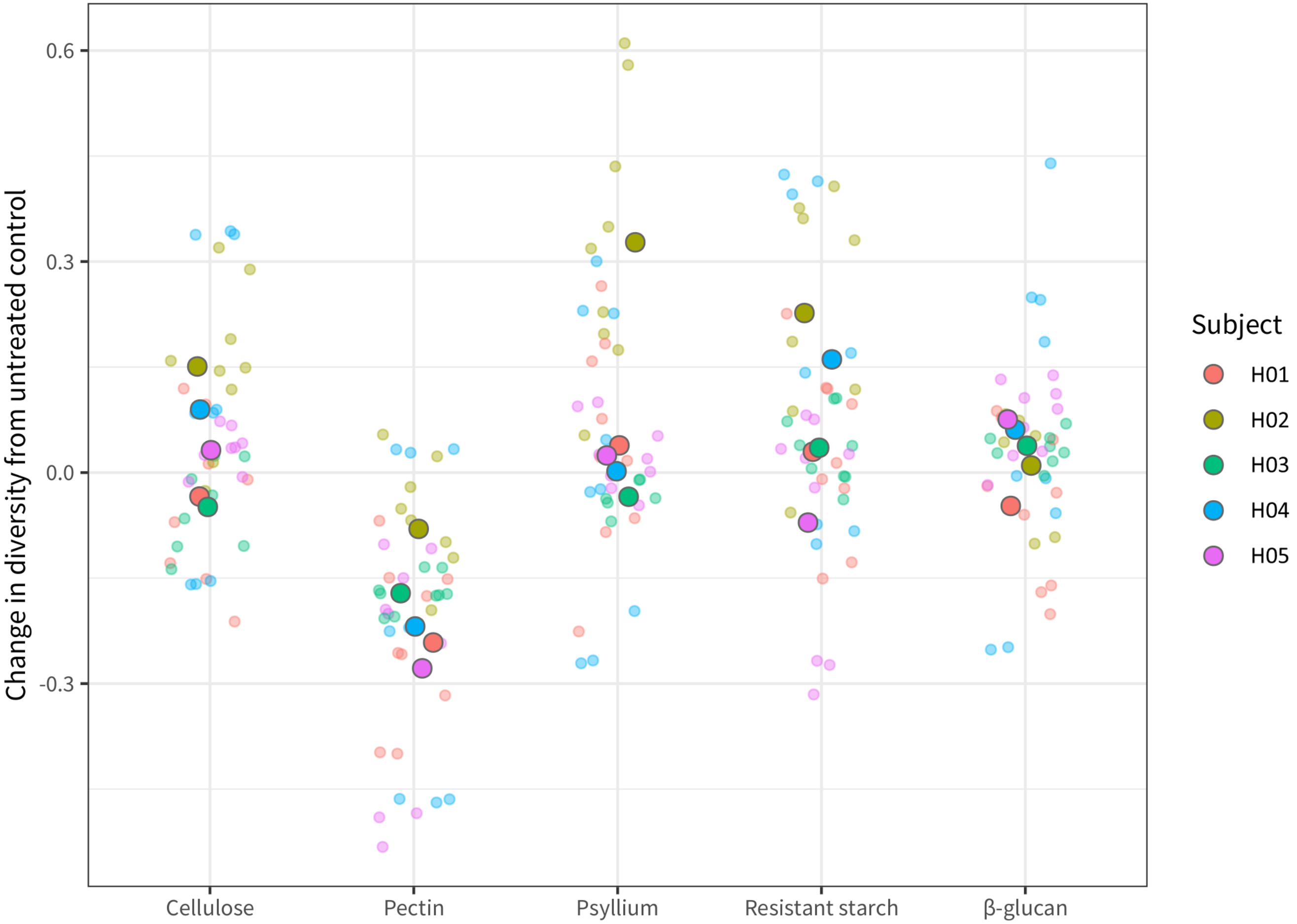

Change in diversity from untreated control
0.6
0.3
0.0
-0.3
Cellulose
Pectin
Psyllium
Resistant starch
β-glucan
Subject
H01
H02
H03
H04
H05
