# Supplementary material for: Individual and Group-Based Effects of In Vitro Fiber Interventions on the Fecal Microbiota
Source: Microorganisms. 2023 Aug 3;11(8):2001. doi: 10.3390/microorganisms11082001 (PMC10459671; doi:10.3390/microorganisms11082001)
Supplement: Supplementary file 1 [file microorganisms-11-02001-s001.zip › microorganisms-2526744-supplementary/SupplementaryFigureS2b.pdf]

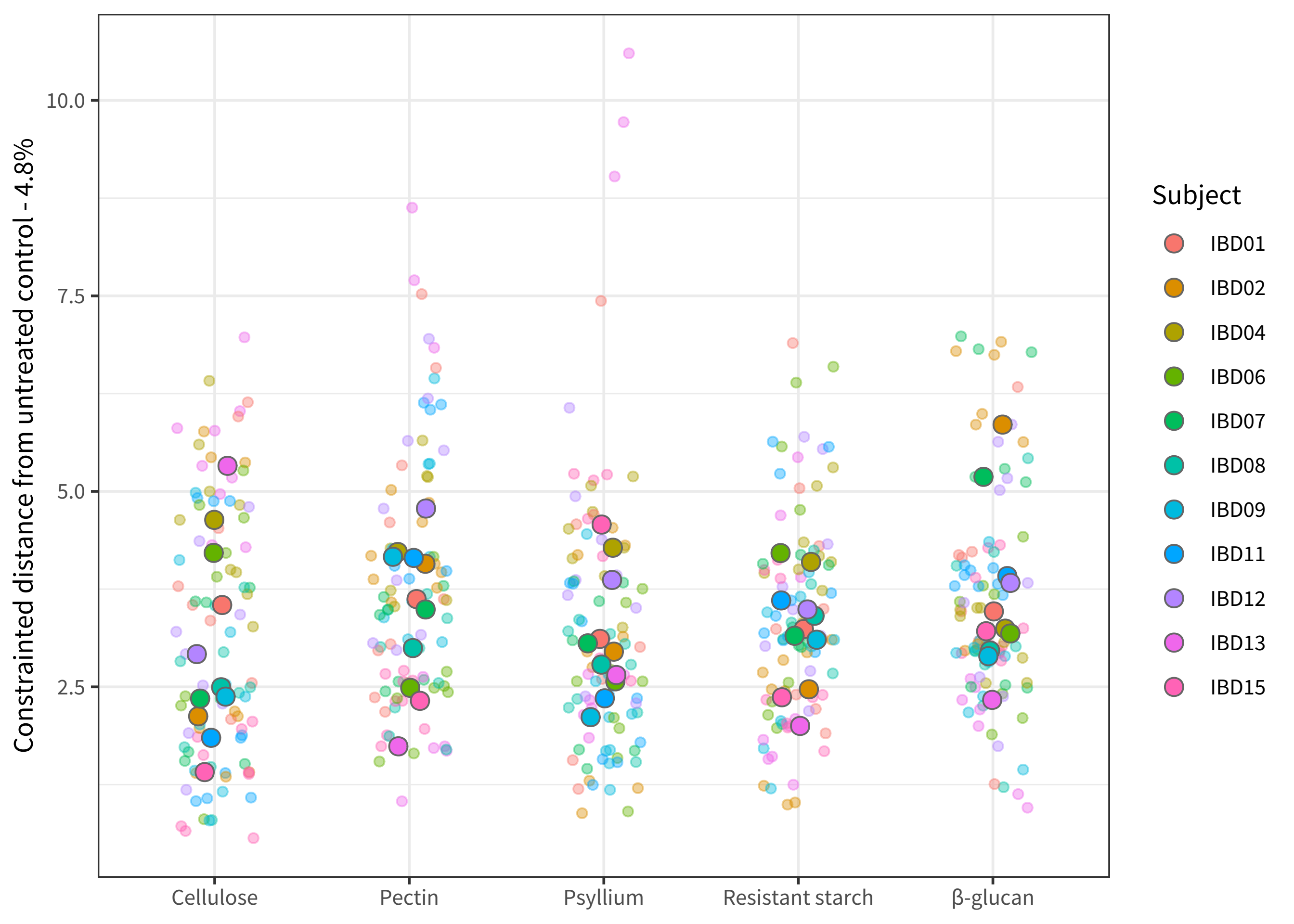
Constrainted distance from untreated control - 4.8%
10.0
7.5
5.0
2.5
Cellulose
Pectin
Psyllium
Resistant starch
β-glucan
Subject
IBD01
IBD02
IBD04
IBD06
IBD07
IBD08
IBD09
IBD11
IBD12
IBD13
IBD15
