# Supplementary figures and images for: Individual and Group-Based Effects of In Vitro Fiber Interventions on the Fecal Microbiota
Source: Microorganisms. 2023 Aug 3;11(8):2001. doi: 10.3390/microorganisms11082001 (PMC10459671; doi:10.3390/microorganisms11082001)

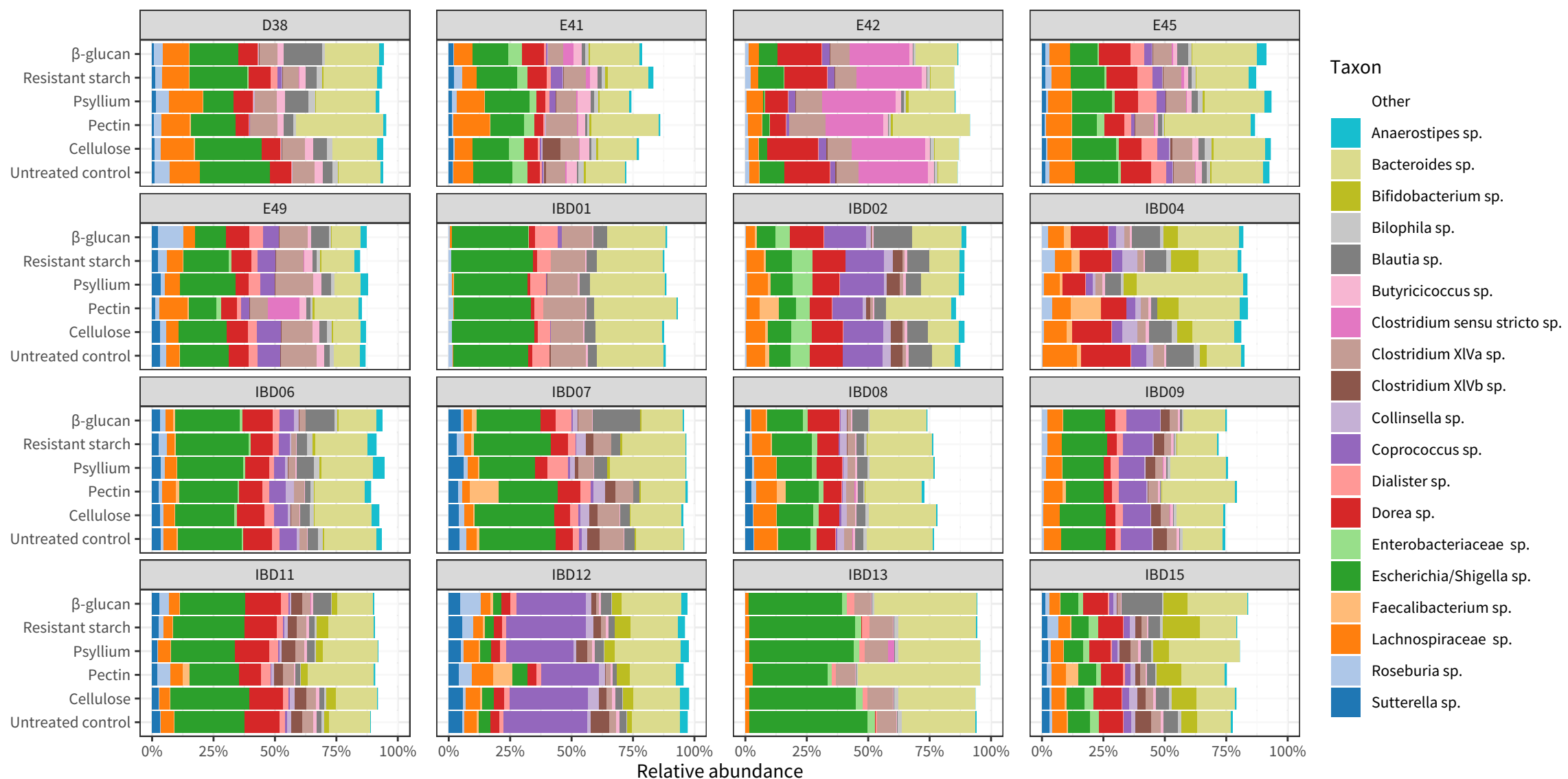

Supplement: Supplementary file 1 [file microorganisms-11-02001-s001.zip › microorganisms-2526744-supplementary/SupplementaryFigure3.pdf]

Constrained distance from untreated control - 9.7%

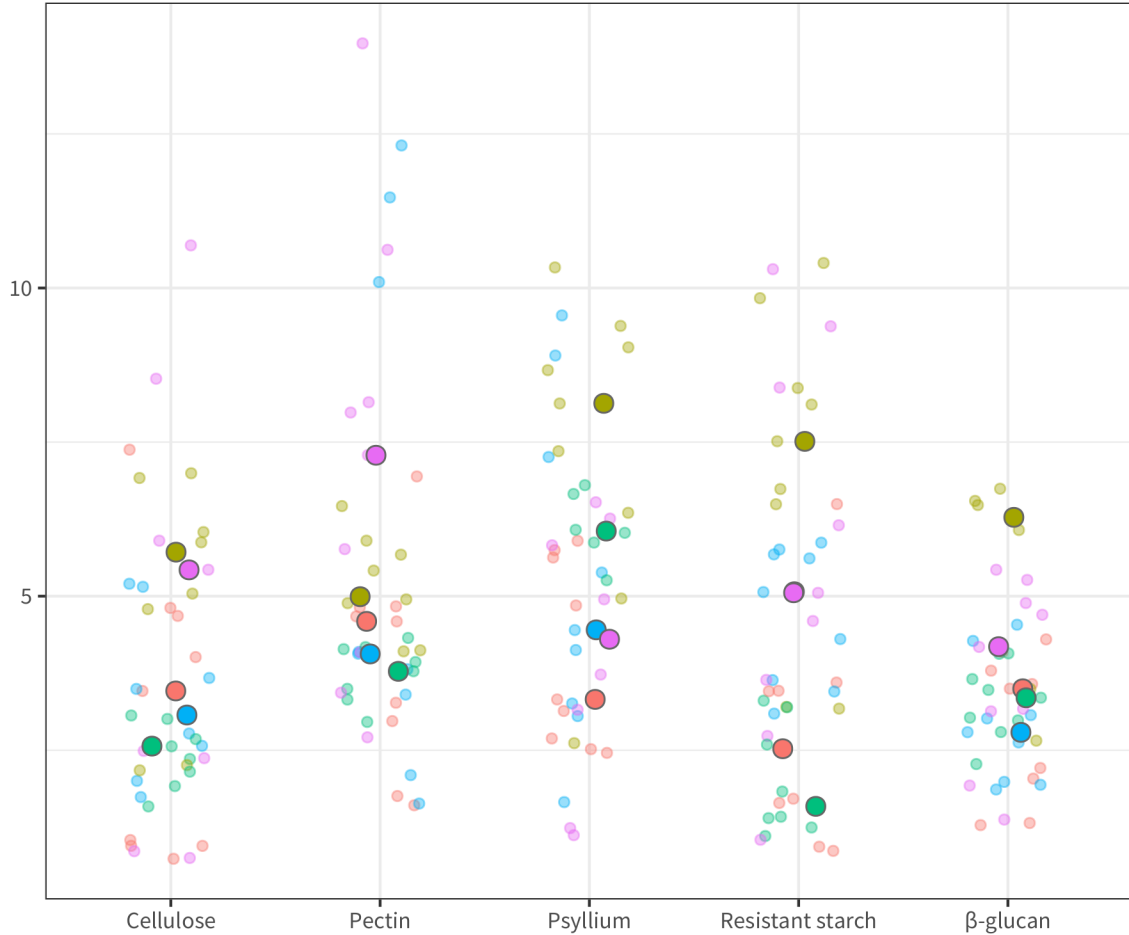

Subject

H01

H02

H03

H04

H05

Supplement: Supplementary file 1 [file microorganisms-11-02001-s001.zip › microorganisms-2526744-supplementary/SupplementaryFigureS2a.pdf]

Contraented distance from control - 18.7%

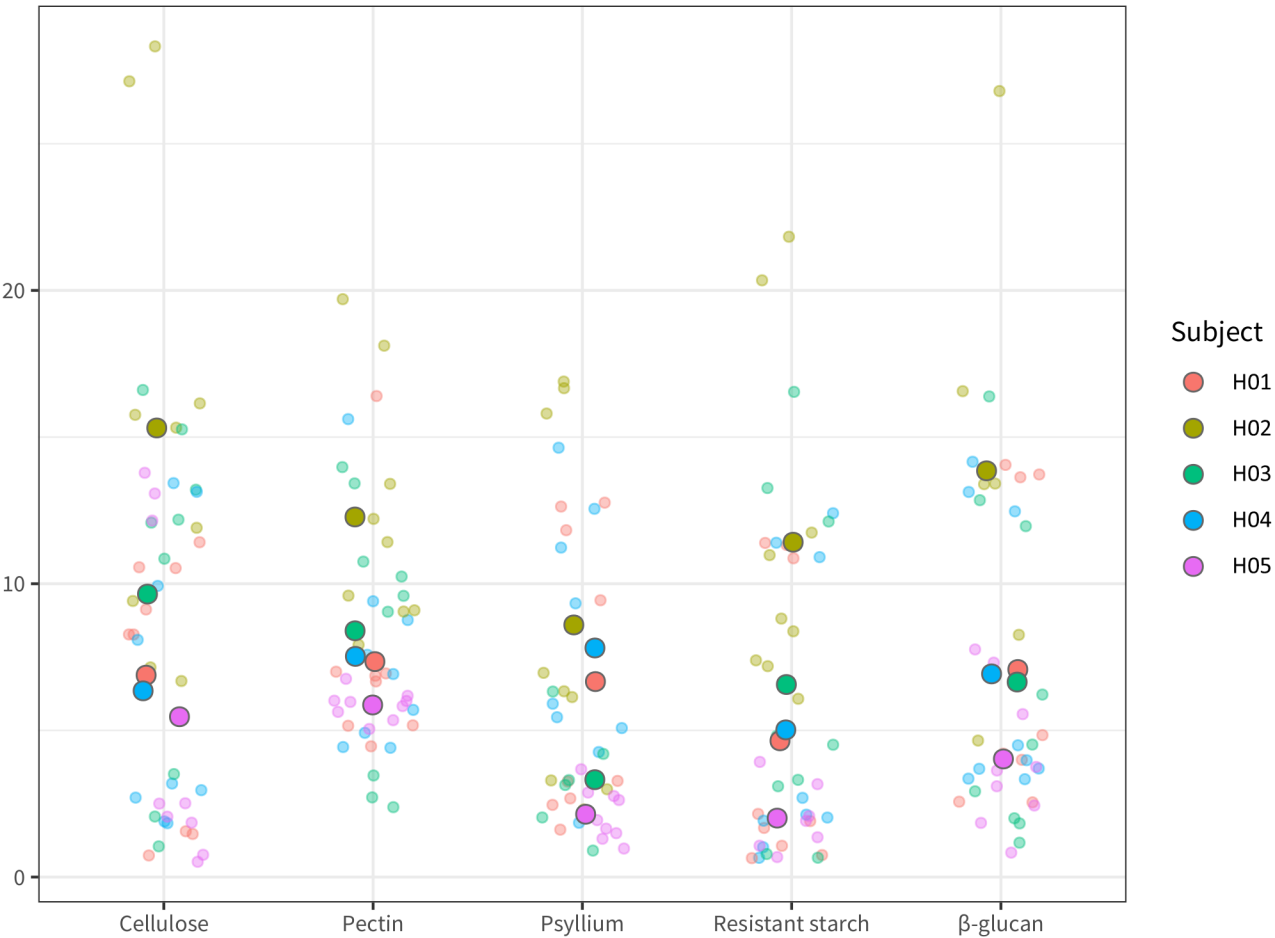

Supplement: Supplementary file 1 [file microorganisms-11-02001-s001.zip › microorganisms-2526744-supplementary/SupplementaryFigureS4a.pdf]
